# Asking for help: An empirical exploration into social grammar

**DOI:** 10.1371/journal.pone.0325598

**Published:** 2025-06-05

**Authors:** Andreas Trotzke, Attila Balla, Hannah Grobauer, Eva Wittenberg

**Affiliations:** 1 Department of Linguistics, University of Konstanz, Konstanz, Germany; 2 Department of Cognitive Science, Central European University, Vienna, Austria; University of Missouri Columbia, UNITED STATES OF AMERICA

## Abstract

This paper explores the interface between linguistic form and social meaning by focusing on correlations between sentence type and the social distance between interlocutors—a central aspect of the social meaning component of politeness. We present a forced-choice experiment with four different groups of speakers (L1 British English speakers, L1 American English speakers, L2 English/German speakers, and L1 German speakers). In this experiment, we manipulated the linguistic form of asking for help along the syntactic dimension of sentence type (declaratives, interrogatives, or imperatives) and recorded the addressee our participants picked for each form (brother, coworker, or stranger). We broaden the empirical picture by going beyond highly conventionalized forms (e.g., *Can you VP?*) and therefore also varying the modal auxiliary verbs (e.g., *Will you VP?*). Based on this comprehensive picture of ways of asking for help, we identify clusters of linguistic forms depending on their felicity in different social scenarios. Our descriptive cluster analysis as well as the statistical comparisons between sentence types indicate that there are systematic correspondences between linguistic form and social meaning across different groups of speakers and languages, and we propose that our empirical data provide a potential starting point for rethinking speech act grammar in terms of ‘social grammar’.

## 1. Introduction

Imagine you are at a train station, you are already late, and you are carrying a heavy suitcase. You could use some help. But how do you ask for it? Many English speakers would probably use the form (1a), a highly conventionalized, standard formulation. Some might also say (1b). But how about the option in (1c)? Intuitively, this option could only be used in a restricted set of social scenarios—for instance, if you are close to the addressee (e.g., your partner or your brother), or if you have institutional power over the person (e.g., your employee). There are of course many more forms to ask for help in a language like English (see [1] for seminal work on the variety of requesting forms), and for most of them it is not so clear when to use them; see (1d) and (1e).

(1) a. Can you help me with this?
  b. Please help me with this!
  c. Help me with this!
  d. Will you help me with this?
  e. You can help me with this.

The examples in (1) illustrate that the same speech act of requesting help can be performed by a variety of linguistic forms. All those different forms do not change the speech act, but they have an impact on the social meaning of the utterance because they feature different degrees of politeness [2–4]. However, while it is clear that using *please* in (1b) is perceived as being more polite than a bare imperative (1c), it is less obvious where exactly the other forms fall on a hierarchy of politeness. Is (1a) more polite than (1b)? And how about (1d)? Is it comparable to (1a) or judged as definitely less polite by native speakers?

Many studies have investigated those questions, but this field of socio-pragmatic research is dominated by work that adopts Searle’s [5] traditional distinction between direct and indirect speech acts (see [1,6,7]; and many others). As a result, Blum-Kulka et al. [1], for instance, only focus explicitly on linguistic form and structure when they talk about examples such as (1b) and (1c). According to their account, those are ‘direct’ request strategies where the linguistic form directly expresses a request. Of course, forms such as (1a), (1d), and (1e) are discussed at length in this type of work too, but only in terms of their interactional function. In other words, since strategies like (1a), (1d), and (1e) are considered ‘indirect’, there is no point in focusing on their linguistic form. According to this functional view, (1a), for instance, can be classified as a ‘query preparatory’ and, based on this functional classification, forms one class with diverse other linguistic forms (e.g., *I was wondering if you would help me with this*; see [1, pp. 278–280]).

In this paper, we try a different path, both conceptually and methodologically. Conceptually, we will challenge the classical ‘indirect’ approach by Searle [5] where forms such as (1a) are categorized as a case of pragmatically inferred politeness (‘if you can, then please do *x*’). Alternatively, some more recent theories have suggested that syntactic forms such as *Can you VP?* are simply a case of ambiguity between an ability question and a polite request [8], without pragmatic inferences at play (see [9] for psycholinguistic evidence). Importantly, there is also socio-pragmatic work by Terkourafi [10–12] suggesting that direct speech-act forms such as the bare imperative (*Help me with this!*) actually represent a special case, and indirect forms such as *Can you VP?* can be considered the natural default. In particular, regarding similar indirect forms of speech Terkourafi [11] has argued that the effect of politeness of utterances such as *Can you VP?* is not based on indirect communication or separate messages communicated by one and the same utterance. Rather, speakers implicitly learn to use different forms in a specific type of social context, and the politeness effect in those cases is thus just a by-product of conventionalization. According to this “renewed understanding of politeness, the crucial distinction is not between direct vs. indirect but between conventionalized vs. non-conventionalized.” [11, p. 15]. Crucially, conventionalization, according to Terkourafi [12, p. 213], is “a matter of degree, and may well vary in different speakers […]. This does not preclude the possibility that a particular expression may be conventionalised in a particular context for virtually all speakers of a particular language, thereby appearing to be a convention of the language.” That is, in cases such as *Can you VP?* those expressions may just be the convention (across all speakers) for performing a request because the concrete linguistic form and the context of requesting co-constitute a ‘frame’ [10], based on the regular co-occurrence of form and pragmatic context.

All in all, the distinction between direct and indirect strategies that has traditionally been applied to the examples in (1) in the literature on speech acts has lost some of its plausibility—and some theories such as Terkourafi’s [10] frame-based approach have already made an attempt at understanding the relationship between linguistic forms and social categories such as politeness in more fundamental ways. This enables us, for the first time, to approach the topic of requesting speech acts from the perspective of linguistic form in a more comprehensive way: We will deal with the various forms in (1) on a par with each other and thus investigate the effect of different sentence types (e.g., interrogative vs. declarative/(1a) vs. (1e)) and of different modal auxiliaries (e.g., *can* and *will* in (1a) vs. (1d)).

The goal is to explore to what extent there is a grammar that determines the mapping between linguistic form and the social meaning component of politeness. By ‘grammar’ we mean a predictable mapping between linguistic form and meaning that takes knowledge about the social relationships between speaker and addressee into account. There is growing interest in recent formal linguistics in social meaning components, both from a syntactic [13–16] and from a semantic perspective [17–19]. However, this work has not yet addressed the differences in social meaning we observe in the linguistic forms in (1).

Methodologically, we will depart from the literature on requests cited above in using a forced-choice comprehension task and not a production task like most of the works cited above do. Since our goal is to systematically manipulate the linguistic forms, the methodology of previous studies seemed to be less adequate for our goals (e.g., discourse completion tasks in [1] and many others).

The paper is structured as follows. In Section 2, we will first sketch the idea of an interface between grammar and social meaning by setting this interface apart from the more traditional concept of a mapping between grammar and speech acts. Based on this distinction, we will introduce a prominent notion of politeness, according to which social distance is a crucial factor in determining the degree of politeness, among other factors. Section 3 presents an experiment with four groups of speakers (L1 British English speakers, L1 American English speakers, L2 English/German speakers, and L1 German speakers) where we manipulated the linguistic form of asking for help along the dimensions of sentence type and modal auxiliaries, and participants had to choose scenarios that varied in social distance for those different linguistic forms. Section 4 summarizes and concludes the paper.

## 2. Background: speech acts, politeness, and social distance

From a formal linguistic perspective, the mapping between linguistic form and the pragmatic domain of speech acts is certainly one of the most prominent topics at the syntax-pragmatics interface [20]. A traditional approach would be to focus on the relevant speech act in a situation like the one described above (i.e., asking for help), and to investigate how the illocutionary force of the speech act is connected to the linguistic forms one could use in a particular situation. However, the classical view of that interface might not be accurate once we approach linguistic forms from the perspective of a concrete communicative situation like asking for help at a train station. Let us see why.

The seminal literature on speech acts has pointed out that changing the linguistic form of an utterance influences how the utterance is interpreted at the level of illocutionary force. In particular, Searle [21, p. 22] gives the following examples where all three utterances are about Sam and smoking, but the different sentence types (declarative, interrogative, and imperative) all express different speech acts: (2a) is an assertion, (2b) is a question, and (2c) performs an order. Note that we have slightly changed these examples because Searle’s [21, p. 22] original example for an order (*Sam smoke habitually!*) sounded quite weird to our contemporary ears.

(2) a. Sam smokes.
  b. Does Sam smoke?
  c. Sam, stop smoking!

Many decades of linguistic research have shown that postulating a direct correspondence between form and speech act is far too simplistic (see [22] for a recent overview of relevant phenomena). For instance, we can perform a question by using the declarative *Sam smokes?* (*Really?*) with rising intonation, or we can as well assert something by using an interrogative such as *Did Sam ever lift a finger to help you?* (meaning: ‘Sam never helped you.’); see [23] for a comprehensive study on the different forms of those non-canonical questions.

While formal grammatical categories such as sentence type thus do not directly translate into speech act type, our driving hypothesis in this paper is that those categories have more systematic correspondences with another dimension of communication, which we refer to as the social meaning dimension. Very broadly, within that dimension we can distinguish between “social meanings grounded in the social relationship between the interlocutors emergent in discourse (e.g., solidarity, politeness) and social meanings that bear on the more durable traits of a speaker identity or personality” [17, pp. 13–14]. In this paper, we focus on politeness and hence the social relationship between interlocutors. For example, we can as well perform the directive speech act in (2c) by saying:

- c. Can you (please) stop smoking, Sam?

By changing both the sentence type and adding a modal auxiliary, the utterance is intuitively more polite than its counterpart in (2c). Since politeness is one of the major aspects of the social relationship between interlocutors, we observe that changes in the linguistic form indeed have an effect on the social meaning dimension of human language.

Utterances like (2c’) have traditionally been analyzed as so-called indirect speech acts [5], and most of the socio-pragmatic literature on the speech act of requesting has adopted this account (see Section 1 above). According to this view, (2c’) is more indirect than its counterpart in (2c) because the speaker is not asking about Sam’s ability to stop smoking (i.e., the literal/‘direct’ reading of the form), but rather the speaker is asking Sam to actually stop smoking. Indirectness in this sense corresponds to a higher degree of politeness (see [24] for the interaction between the two concepts).

In the context of our study, it is interesting that fundamental work such as Blum-Kulka’s [24] large-scale study also makes very clear that it matters a lot at whom a relevant speech act is directed, and that therefore the social dimension of meaning has an impact on the linguistic forms we use to perform speech acts. For instance, you can keep the information asked for constant in the examples in (3), but still observe a significant difference in the linguistic forms you would use to address the question to either a child or an elderly lady, for instance:

(3) a. How old are you? [✔a child ✘elderly lady]
  b. May I ask how old you are? [✔a child ✔elderly lady]

The social dimension involved in linguistic forms such as (3) and hence their different degrees of politeness can be further decomposed by adopting a calculation of the weightiness of face-threatening acts proposed by Brown & Levinson in their seminal work on politeness [2]. In particular, the weightiness (*W*) of a face-threatening act (*x*) like asking a question such as (3) can be calculated as follows [2, p. 76]:

(4) W*x* = D(S,H) + P(H,S) + R*x*

According to this formula, the weightiness is the sum of the distance (*D*) between S(peaker) and H(earer), the power (*P*) that H has over S, and the degree to which the respective face-threatening act is considered an imposition in the respective culture (R*x*). According to the politeness theory by Brown & Levinson [2], those three factors determine the level of politeness with which the face-threatening act will be communicated. In our example (3) above, the social distance D(S,H)—given standard assumptions about the difference between asking a child and an elderly lady about their age in Western societies—differs considerably, and that is why a speaker would choose different levels of politeness (i.e., different linguistic forms) for performing the relevant speech act.

In this paper, we will focus on this component of the social distance D(S,H) between speaker and addressee. However, we hasten to add that the other two dimensions (power and imposition) have interesting reflexes in the linguistic forms we use to perform speech acts as well. For instance, the differences in power constellations between a threat and a request is represented by very different expressions (5), in languages like German even by dedicated particle elements (5’); see [25] and [26]:

(5) a. Do you hear me? Give me the money back! [threat]
  b. Please give me the money back! [request]
(5’) a. Gib mir bloß das Geld zurück! [threat]
  give
    me prt the money
    back
  ‘Do you hear me? Give me the money back!’
  b. Gib mir bitte das Geld zurück! [request]
give
  me prt the money
  back
‘Please give me the money back!’

The differences in imposition certainly feature a higher degree of cultural and contextual variation, but in this context too we find relevant reflexes in linguistic forms. Look at the following cases. If we assume that asking for information about one’s bank account balance is considered a higher imposition than asking about the time, then this is the reason for why (6b) would be pragmatically deviant:

(6) a. Would you mind perhaps just telling me how much money is in your bank account?
  b. #Would you mind perhaps just telling me what time it is?

Therefore, even if there are good reasons to assume that the dimensions of power and imposition play an important role in our choices of linguistic forms as well, those two dimensions are harder to test and manipulate. In particular, (6b) might be perfectly fine if the speaker is just a very careful or even anxious person—and (5a)/(5’a) can in fact also be uttered by an employee who can threaten her boss in a situation where they have known each other for many years. However, the difference in social distance between a brother and a stranger, for instance, is probably less context-dependent for speakers coming from the same language and culture. We thus hypothesize that social distance is a component of politeness that can also be tested in a forced-choice experiment.

In what follows, we therefore focus on social distance, and in the next section, we present an experiment that explored the correlations between different linguistic forms of asking for help and different scenarios that varied in social distance. The main goal of this empirical work is to identify clusters of linguistic forms depending on their felicity in different social scenarios and, based on those clusters, to zoom in on the core syntactic categories of sentence types and their effect on the social meaning of an utterance used for performing a request—in our case: asking for help at a train station.

## 3. Clusters and syntactic categories of social grammar

In this section, we report the results of a forced-choice experiment. We manipulated the linguistic forms of performing a request, measuring which interlocutors our participants thought the utterance was directed at. Our study is asking the following two general questions:

- Are the number and the composition of **clusters** uniform across different groups of speakers?
- Does the **category of sentence type** have the same effect across different groups of speakers?

To answer those two questions, we present data from four groups of speakers (L1 British English speakers, L1 American English speakers, L2 English/German speakers, and L1 German speakers). As already indicated in Section 1, we did not take into account any additional measures of socio-pragmatic differences in politeness cultures between different varieties of English [27–30]. Rather, we adopted a form-oriented perspective and thus solely focused on morphosyntactic differences—and for all four groups of speakers, we manipulated the linguistic form of asking for help along the same linguistic dimensions and along the same dimensions of social distance. In what follows, we detail the relevant materials. Analysis scripts and data for all experiments can be found in a public repository at https://osf.io/fvra9.

### 3.1 Methods

#### Materials.

In our experiment, we focus on the speech act of requesting (in a situation where someone is asking for help at a train station). The different forms of requests studied in this experiment were designed along the three major sentence types declarative, interrogative, and imperative. In addition, we have broadened the empirical picture by also including a variation of the modal auxiliary verbs to go beyond highly conventionalized forms such as *Can you VP?* Since the imperative does not allow for this kind of lexical variation, we chose to vary this sentence type by either using or not using the particle *please* (German ‘bitte’). Regarding the modal verbs, we have chosen four different modalities (*can*, *will*, *would*, *must*) and thus excluded further—also highly frequent—options such as *could* (see [30, pp. 82–83] for an overview of possible forms in both British and American English). We did not want to use two instances of the same modality (e.g., *can* and *could* for ‘ability’) and thus had to be selective with our items, conceding that our list is not representing all conceivable and common options for performing a request. This resulted in ten different items, which are shown in Table 1 for the two languages, English and German.

**Table 1:** Overview of experimental items in both English and German.

Sentence type	Modal verb/particle	English form	German form
interrogative	*can*	*Can you help me with this?*	*Kannst du mir damit helfen?*
interrogative	*will*	*Will you help me with this?*	*Wirst du mir damit helfen?*
interrogative	*would*	*Would you like to help me with this?*	*Würdest du mir gerne damit helfen?*
interrogative	*must*	*You must help me with this, don’t you?*	*Musst du mir damit nicht helfen?*
declarative	*can*	*You can help me with this.*	*Du kannst mir damit helfen.*
declarative	*will*	*You will help me with this.*	*Du wirst mir damit helfen.*
declarative	*would*	*I would love your help with this.*	*Ich hätte gerne deine Hilfe damit.*
declarative	*must*	*You must help me with this.*	*Du musst mir damit helfen.*
imperative	∅	*Help me with this!*	*Hilf mir damit!*
imperative	*particle*	*Please help me with this!*	*Bitte hilf mir damit!*

The items were presented in written form, and we acknowledge that we have thus disregarded the phonological dimension that might also influence the way in which the utterances are interpreted significantly. For instance, a reviewer pointed out to us that native speakers of English might perceive a big difference in pronouncing the modal verb *can* with a full or a reduced vowel: The reduction would be fine in informal speech and thus be perceived as ‘less distant’ by native speakers. Our study presents a first exploration asking whether and, if so, which broad patterns exist at the interface of linguistic form and the social meaning of an utterance used for performing a request. We hope that our data can inform follow-up studies that manipulate relevant utterances along the dimension of phonological variation.

#### Participants.

The study was approved by the Psychological Research Ethics Board of the Central European University and conducted in accordance with institutional guidelines, with informed written consent obtained from all participants. Data were collected between March 20^th^, 2024, and July 30^th^, 2024.

Ninety-five native English speakers from the UK (male: 26; average age: 40; range: 21–70), 100 native English speakers from the US (male: 48; average age: 38; range: 18–77), and 101 native speakers of German (male: 50; average age: 35; range: 18–77) participated in the experiment. All those speakers were recruited through the crowdsourcing platform Prolific and compensated for participation. We did not exclude any participants, and the study took on average 3 minutes to complete. For L2 English speakers, we recruited 34 native German speakers in an in-person setting at the University of Cologne (female: 30; average age: 23; range: 19–30; for demographic and linguistic details, see Appendix).

#### Procedure.

Participants of the four groups of speakers were presented with the following short scenario:

*Tom is a young guy in his late twenties, and he is at the train station and carries two heavy suitcases. To board the train in time, he is in urgent need of help with loading the suitcases onto the train. Tom says to the person next to him…*

For the 34 German participants who were tested on the English items (i.e., L2 English speakers), the scenario was given in English as well. For the 101 German participants who were tested on the corresponding German items, the same scenario was given in German (German translation: “Tom ist ein junger Mann Anfang zwanzig. Er ist am Bahnhof und muss zwei sehr schwere Koffer tragen. Um rechtzeitig in den Zug einzusteigen, braucht er dringend Hilfe, um die Koffer in den Zug zu heben. Tom sagt zu der Person, die neben ihm steht: …”).

**Fig 1:**
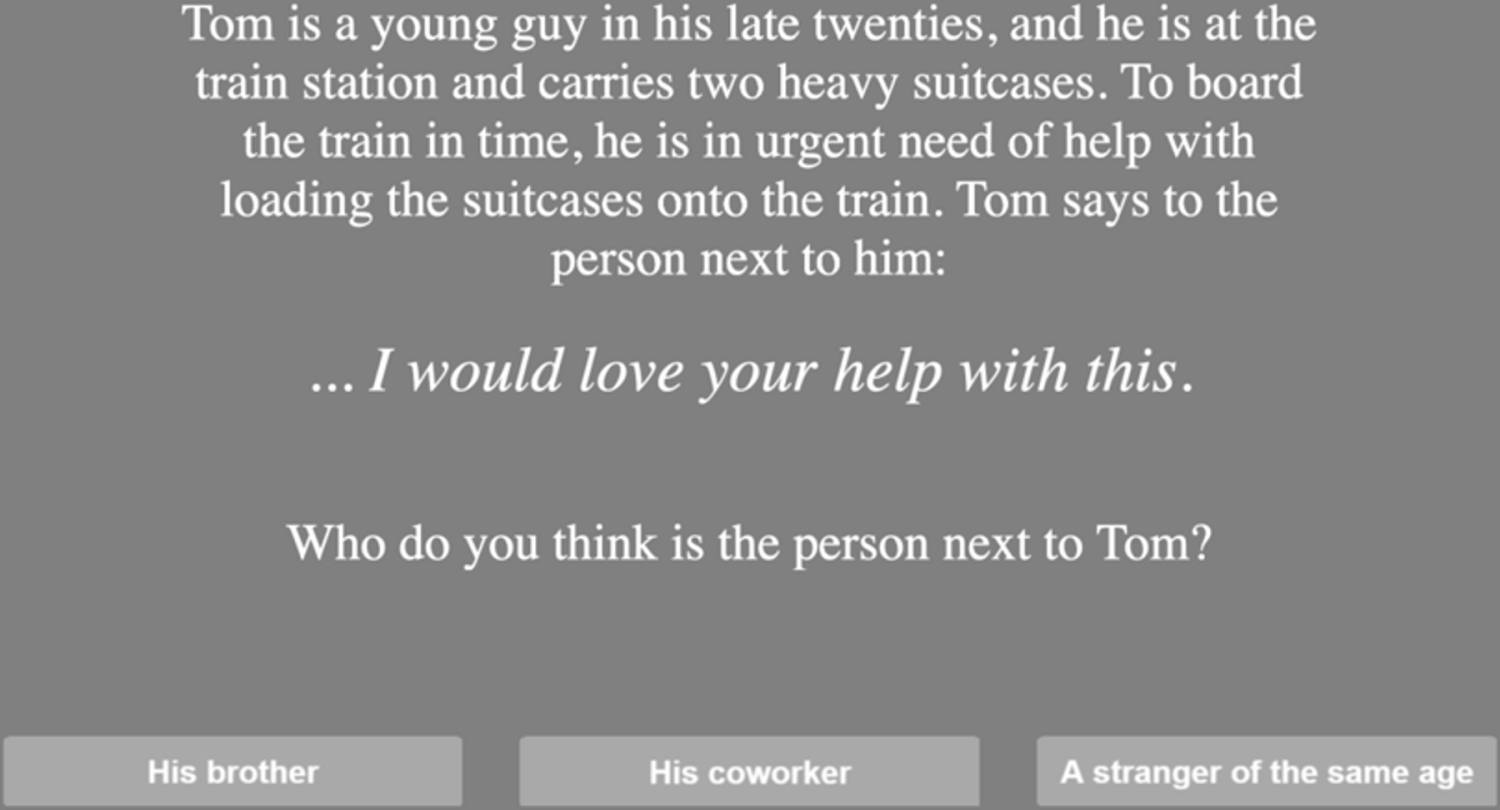
Screenshot of a trial as presented to English online participants.

The scenario was followed by one of the ten different forms of performing a request given above (Table 1). In a forced-choice task, participants then indicated to whom they believed Tom’s request for help was directed. Participants could choose from three options: his brother, his coworker, or a stranger of the same age (see Fig 1). In terms of the decomposition of politeness by Brown & Levinson [2], we thus kept the imposition component constant (by always referring to the same action asked for) and left the power constellation open by not mentioning anything about power relations (see Section 2 for discussion). Accordingly, we only manipulated the social distance component of politeness.

Crucially, in the case of German speakers tested on German it was clarified to German participants prior to the study that the conversation takes place between young people of the same age, who tend to use the informal ‘you’ (*du*), and not the formal ‘you’ (*Sie*) among each other [31,32]. Accordingly, as shown in Table 1 above, we only used the informal ‘you’ (*du*) in our items because we were interested in the effect of sentence types and varying the modal auxiliaries, and not in the additional effect of different pronouns. This had the methodological advantage that we could keep the contexts constant across the English and German speakers: While (young) people of the same age can use informal ‘you’ (*du*) for all three options (brother, coworker, and stranger), no one would use formal ‘you’ (*Sie*) for addressing their brother. In a sense, informal ‘you’ (*du*) can thus be seen as the unmarked pronominal form, given our three choices and the context of young people of the same age talking to each other.

Based on the data collected for all four groups of speakers on the items and scenarios sketched above, we conducted two types of analysis: a cluster analysis asking whether we find the same number and composition of clusters across the different groups of speakers (Section 3.2.1) and an analysis focusing on the effect of the category sentence type on the judgments across the different groups of speakers (Section 3.2.2). We start with presenting our cluster analysis for the four groups of speakers.

### 3.2 Analysis and results

#### 3.2.1 *Cluster analysis.*

For the judgments of all four groups of speakers, we conducted *k*-means cluster analyses. Data processing and statistical analyses were carried out using R Studio (Posit Team 2024). We performed a hierarchical cluster analysis on participants’ selections to identify linguistic forms that clustered together along the three choices of social distance (brother, coworker, or stranger). Hierarchical cluster analysis consists of a set of techniques to explore multivariate datasets and understand their underlying structure. It is particularly well suited for examining the correlational patterns among different measures [33], and it is thus an appropriate analysis to address our first main question, namely whether the number and the composition of clusters is similar across different groups of speakers.

**Fig 2:**
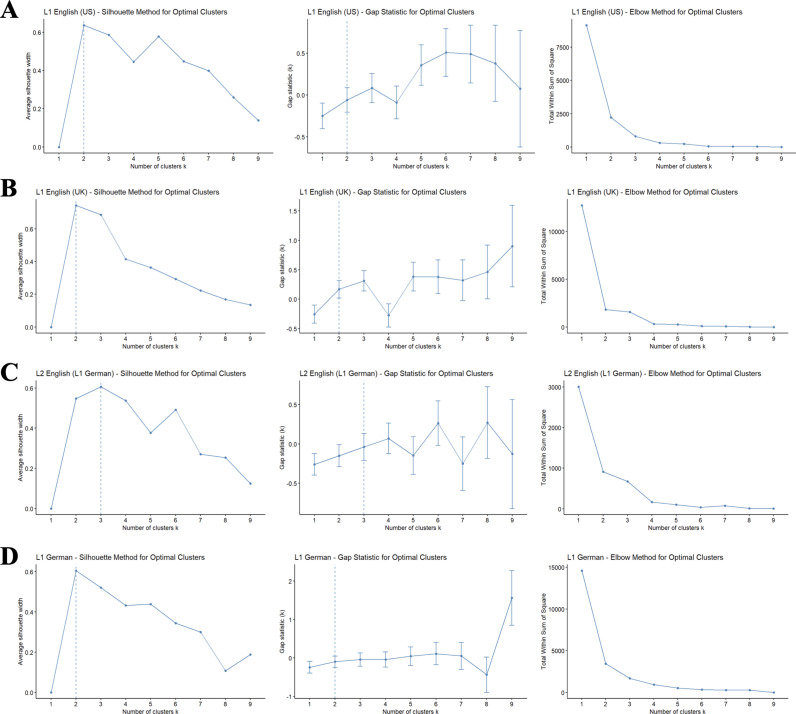
Measures to determine number of clusters, for each version of the experiment: A. L1 English (US), B. L1 English (UK), C. L2 English (L1 German), D. L1 German. The left columns show results of the Silhouette method, the middle column the gap statistic, and the right column shows results of the Elbow method.

To determine the optimal number of clusters for each experiment version (L1 English/UK, L1 English/US, L2 English/German L1, and L1 German), we applied the elbow method, the average silhouette method, and the gap statistic [34]. We used Ward’s method as the linkage criterion and Euclidean squared distance as the distance measure. Fig 2 shows the results of the cluster-finding procedures. Taken together, each of these methods suggested that *k = 2* was the optimal number of clusters for each dataset; in the L2 English (German L1) version, the optimal number of clusters was *k = 3* according to two out of three methods (silhouette and gap statistic tests), and *k = 2* according to the elbow method.

To further ensure adequacy of the clustering, we conducted Mann-Whitney U tests on the brother, coworker, and stranger scores. For the three-cluster solution in the L2 English (L1 German) group, none of the follow-up comparisons were significant, possibly due to much lower power (*n* = 34); therefore, we decided to use a two-cluster solution uniformly across experimental versions. Except for one, in all the comparisons, there was a significant difference between the two clusters (2.193 < *z* < 2.611, 0.011 < *p* < 0.014). The exception was the L2 English (L1 German) dataset’s comparison of coworker scores, where we did not find any significant difference between the two clusters, again possibly due to lower power (*z* = 1.36, *p* = 0.21).

**Fig 3:**
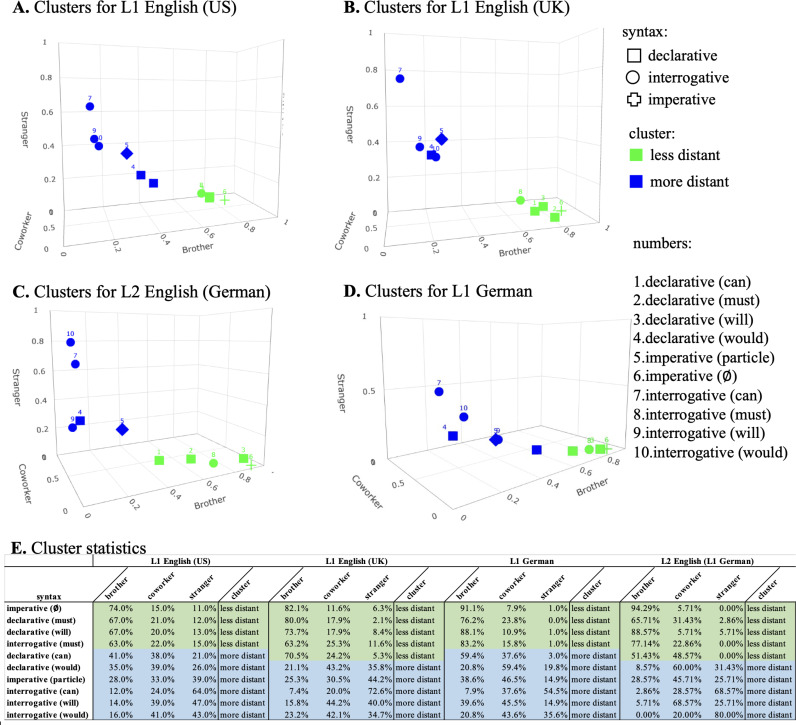
Clusters for each version of the experiment: A. L1 English (US), B. L1 English (UK), C. L2 English (L1 German), D. L1 German. Shapes indicate sentence type, color the resulting cluster, and individual numbers represent each of the ten linguistic forms (see Table 1 in Section 3.1). E: Cluster statistics for each sentence; color codes correspond to less distant (green) and more distant (blue) clusters in A-D.

Fig 3 shows the results of clustering for each experiment version (L1 English/UK, L1 English/US, L2 English/German L1, and L1 German); ‘less distant’ and ‘more distant’ refers to the social distance to the interlocutor measured in our experiment by means of the three choices ‘brother’, ‘coworker’, and ‘stranger’.

#### 3.2.2 *Category analysis.*

For an analysis that focuses on the effect of the category sentence type on the judgments across the different groups of speakers, we first coded choices on a scale from ‘most distant’ to ‘least distant’ as ‘1’ (stranger), ‘0’ (coworker), and ‘-1’ (brother). For statistical analyses, we transformed the data into a binomial range and performed *t*-tests, corrected for multiple comparisons using Bonferroni corrections.

**Fig 4:**
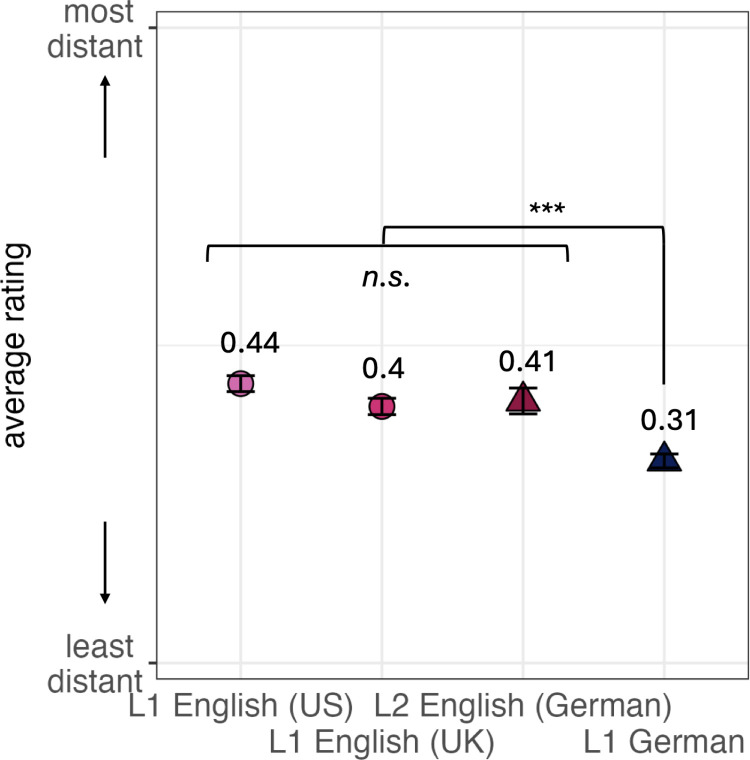
Overall choices per language group. Numbers and symbols indicate mean scores on transformed data; whiskers show the Standard Error of the mean.

Fig 4 illustrates that US English speakers were most likely to choose distant interlocutors across items. However, they were not significantly different form the other English participants, including German L2 speakers (all *p*s > .22). However, the L1 German group chose significantly less distant interlocutors than any other group (all *p*s < .001).

**Fig 5:**
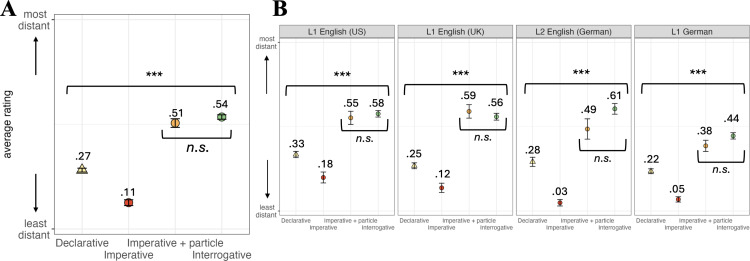
Overall choices by sentence type across language versions (A) and within each language version (B). Numbers and symbols indicate mean scores on transformed data; whiskers show the Standard Error of the mean.

We also conducted comparisons within and across experiment versions to answer our second main question (see above), namely whether the **category of sentence type** elicits similar choices across different groups of speakers. Fig 5-A illustrates that indeed across versions, declaratives (yellow) and imperatives without the particle *please* (red) were used with interlocutors of smallest social distance. Imperatives with the particle (orange) were almost as polite as the interrogative (green), which was judged to be used with the most distant interlocutor. This pattern is reflected in pairwise *t*-tests, which differed significantly between all conditions (all *p*s < .001), except the imperative with particle and the interrogative (*p* > .99).

Importantly, this pattern was found in each experiment version (i.e., across the different groups of speakers; Fig 5-B). That is, all *p*s < .001, except the comparisons between imperatives with particle and interrogatives, where all *p*s > .34).

### 3.3 General discussion

In the previous section, we presented the results of a forced-choice experiment that we conducted with four different groups of speakers: L1 British English speakers, L1 American English speakers, L2 English/German speakers, and L1 German speakers. In this experiment, we manipulated the linguistic forms of performing a request where someone is asking for help at a train station. We asked participants to choose from three choices which interlocutors the different linguistic forms were directed at: brother, coworker, and stranger. Given our theoretical discussion of the notion of politeness in Section 2 above, we thus manipulated the social-distance component of politeness.

The goal of our empirical study was to address the following two general questions about the set of utterances tested in our experiment:

- Are the number and the composition of **clusters** uniform across different groups of speakers?
- Does the **category of sentence type** have the same effect across different groups of speakers?

As for the first question, the results of our cluster analysis reported in Section 3.2.1 show that both the number and the composition of clusters is remarkably similar across the different groups of speakers. In particular, we concluded that a set of measuring techniques indicate that *k = 2* was the optimal number of clusters for each group, and we have explained (and run additional tests) for the L2 English (German L1) group where some deviations from that pattern is probably due to the lower number of participants (and the resulting lower power).

Regarding the composition of the clusters, we also see only small differences. Specifically, only one item varied across the groups of speakers: The declarative *You can help me with this* is preferably used by L1 American English speakers and by L1 German speakers (German: *Du kannst mir damit helfen*) for socially more distant addressees. By contrast, L1 British English speakers and L2 English (L1 German) speakers would use it for socially less distant addressees.

We can only speculate why that might be the case. There might be cultural differences between US and UK speakers in that regard, and this difference might have been carried over to L2 English (L1 German) speakers because British English is the dominant version L2 learners in Germany are confronted with in the school and education systems. But again, our data do not tell us anything about such cultural differences, and we can only speculate. Except for this one example, we see that overall the composition of clusters is notably uniform across the different groups of speakers.

Crucially, what the four datasets have in common is that there is a clear tendency of interrogative syntax (no matter which form of modality is used) to be judged as more appropriate with more distant addressees and of declarative syntax (again, no matter which form of modality is used) to be more appropriate with less distant addressees—which brings us to our second question whether the category of sentence type has the same effect across different groups of speakers. In this context, we first noted that irrespective of sentence type, the L1 German group sticks out because they chose significantly less distant interlocutors than any other group. We hypothesize that this might be the case because we asked German participants to conceptualize all three choices of interlocutors (brother, coworker, stranger) as persons of the same age that could be addressed by informal ‘you’ (German *du*); see Section 3.1 above for theoretical reasons for this methodological choice.

Abstracting away from this difference, we see that the category of sentence type has the same effects on the politeness component of social distance across the different groups of speakers. More specifically, declaratives and imperatives without the particle *please* were used with interlocutors of smallest social distance, whereas imperatives with the particle pattern with the interrogative in being preferred for the most distant interlocutor. In fact, additional statistical tests show that the latter two choices (imperative + *please* and interrogative) were indistinguishable because there was no significant difference between them. This result is noteworthy because our set of items also varied the choices of modal verbs in interrogatives, including items as diverse as *can* (ability) and *must* (deontic) for instance (see Section 3.1). However, even those differences could not diminish the strong effect of sentence type (in this case: the interrogative) on the social meaning component tested by our study. The same holds, vice versa, for the effect of declarative syntax, which patterns with the bare imperative and overrides potential differences due to the use of different modal verbs.

All in all, this points to a scale of politeness between the lowest point expressed by the bare imperative (which was expected and confirmed by our study) and declarative syntax (which tends to fall into the same cluster as the bare imperative) on the one hand—and on the other end of the scale the imperative with *please* (as expected) and the highly conventionalized *Can you VP* plus interrogative syntax more generally. Importantly, the number and composition of the clusters across the groups of speakers indicate that there are systematic correspondences between linguistic form and social meaning, also across languages (English and German) and across speakers with different native languages (L1 and L2 English). Since the form-function mapping is quite robust in the domain of social meaning measured in our experiment, we take this as empirical support for the idea of a (native) speaker’s grammar in that social meaning domain. In the last section, we will point out some more general consequences of this idea.

## 4. Conclusion

The question of how humans process social information is one of the most debated topics in cognitive science [35]. Our study has focused on the morphosyntactic reflexes of processing social information by investigating how different forms of requests are mapped to the social distance between the speaker and an imaginary interlocutor. Regarding the variable of social distance, we acknowledge that previous research, particularly on politeness, has repeatedly pointed out over the years that “there is no consensus […] among either linguists or psychologists as to reliable ways of distinguishing close and distant relationships […], and there is considerable inconsistency among studies in terminological usage” [36, p. 21]. Social distance is conceptualized quite differently across cultures, but also across other dimensions of variation such as gender (see [37] and for recent empirical work [38]). This might be the main reason for the terminological inconsistencies found in the literature—different studies use the term social distance in divergent ways because they are essentially dealing with distinct phenomena when investigating distance dimensions in different cultures or social groups. In our study, we have not tested the concepts of ‘brother’, ‘coworker’, or ‘stranger’ across the different speaker populations and separated from the linguistic choices speakers had to judge in our comprehension task. Our driving hypothesis which we call ‘social grammar’ predicts that differences in the conceptualization of social distances across populations would be reflected in the grammatical choices speakers make in typologically very similar languages such as American English, British English, and German. We thus turn the tables, as it were, on most of the existing literature on the topic by looking at social distance dimensions from the perspective of linguistic form. As already pointed out at the outset of our article, there is a long tradition to investigate the variety of requesting forms in different languages (see [1]). However, most of those studies have used production tasks and did not manipulate the forms used for requests based on morphosyntactic criteria. We have departed from this tradition and used a forced-choice comprehension task where we could systematically manipulate linguistic form.

Most of the previous studies have not taken this form-oriented path because many of them adopted the Searlean distinction [5] between direct speech acts (e.g., the imperative for requests) and indirect forms (e.g., *Can you VP?* for requests). This position has also been articulated in psychological research where direct forms are considered a cognitive baseline, and the speaker must have certain rational reasons to choose other and more ‘indirect’ forms (e.g., [39—40]). One notable exception in this line of research, already mentioned in our introduction, is Terkourafi’s work on indirect speech [10–12]. In her work, she looks at the distinction direct vs. indirect speech acts from a reversed perspective: “given the ever-present dynamics of politeness and face, why would a rational communicator ever choose to be direct?” [41, p. 2861]—and indeed, direct forms such as the bare imperative, for instance, seem to be rare in actual speech [42]. There is also more recent work in the psycholinguistic literature that doubts the traditional account of dealing with direct forms as the baseline from a processing perspective (e.g., [9] and [43]).

The point already highlighted by Terkourafi’s studies and recently formulated by Trotzke in syntactic work [16] is that once we acknowledge that syntactic choices have systematic consequences for the social meaning component of sentences, we can re-define canonical versions of a speech act as those versions of a speech act that are *socio-pragmatically* unmarked, meaning that they can be used in a less restricted set of social situations. For our cases investigated in the experiment above, this means that the bare imperative (for many: the ‘direct’ speech act) can only be used for less distant (e.g., brother) and not for more distant interlocutors. The interrogative, however, which many call the ‘indirect’ version, can be used for more distant interlocutors as well, but, crucially, that means that it can also be used for less distant ones. This is because you can be polite to anyone (e.g., both to your brother and to a stranger—your brother would be happy). However, this does not work the other way around: It is probably fine to use fewer politeness cues with your brother, but you cannot do without such cues when talking to a stranger, at least if you care about achieving your respective illocutionary goals. In sum, this means that interrogatives, in the case of requests, form the canonical (i.e., less restricted) versions of that speech act, while imperatives without *please* as well as declaratives are the marked and non-canonical ones.

While we all know (and were probably taught already by our parents and/or teachers) that you cannot be pragmatically/socially ‘wrong’ if you always use *please*, the rules are less clear for grammatical categories such as sentence types. Especially in the context of learning and teaching second or third languages, requests are one of the main phenomena in the socio-pragmatic domain [44–48]. Our study is a first attempt to uncover the grammatical rules that go beyond using *please* in that domain, and we consider this a promising path because our results clearly indicate that there is systematicity in the mapping between core syntactic categories and the processing of social relationships between speaker and addressee.

## Appendix: L2 speakers of English

For German native speakers who participated as L2 English speakers, we collected information about first languages, years of studying English, occasions and hours per week using English, and time spent in English-speaking foreign countries. All but one participant named German as their native language. Five participants mentioned multiple native languages.

**Fig 6:**
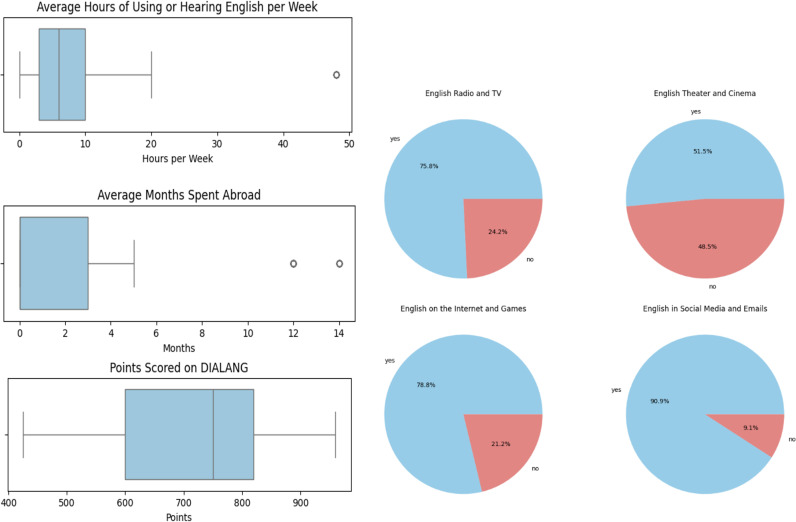
Left: Language background information on German participants: time spent using English per week (top); time spent abroad in English-speaking countries (middle); points scored on the DIALANG English test (bottom). **Right: Contexts in** **which our participants use English**.

Fig 6 gives an overview of the most important indicators of L2 English speakers’ linguistic background. The average number of years spent learning English for our L2 participants was 11 years, and they use English approximately 7 and a half hours per week on average, particularly during online activity. German participants who spent time in English-speaking foreign countries did so for a maximum amount of 14 months. 23 participants did not spend longer periods in English-speaking countries.

All participants also completed the DIALANG vocabulary test, an excellent quick indicator of level of attainment [49—50]. Importantly, the average DIALANG score was 709 points, with a range between 425 and 960 points, which indicates that all speakers commanded a level of English with a good to extensive vocabulary and reliable communicative skills in English.
